# High Frequency of COVID-19 Vaccine Hesitancy among Canadians Immunized for Influenza: A Cross-Sectional Survey

**DOI:** 10.3390/vaccines10091514

**Published:** 2022-09-10

**Authors:** Valeria Valerio, Emmanouil Rampakakis, Theodoros P. Zanos, Todd J. Levy, Hao Cheng Shen, Emily G. McDonald, Charles Frenette, Sasha Bernatsky, Marie Hudson, Brian J. Ward, Inés Colmegna

**Affiliations:** 1 The Research Institute of the McGill University Health Centre (MUHC), Montreal, QC H4A 3J1, Canada; 2 Department of Pediatrics, McGill University, Montreal, QC H4A 3J1, Canada; 3 Institute of Bioelectronic Medicine, Feinstein Institutes for Medical Research, Northwell Health, Manhasset, NY 11030, USA; 4 Institute of Health Systems Science, Feinstein Institutes for Medical Research, Northwell Health, Manhasset, NY 11030, USA; 5 Donald and Barbara Zucker School of Medicine at Hofstra/Northwell, Northwell Health, Hempstead, NY 11549, USA; 6 Division of General Internal Medicine, Department of Medicine, University of Montreal, Montreal, QC H3T 1J4, Canada; 7 Division of General Internal Medicine, Department of Medicine, The Research Institute of the McGill University Health Centre (MUHC), Montreal, QC H4A 3J1, Canada; 8 Division of Infectious Diseases, Department of Medicine, The Research Institute of the McGill University Health Centre (MUHC), Montreal, QC H4A 3J1, Canada; 9 Division of Rheumatology, Department of Medicine, The Research Institute of the McGill University Health Centre (MUHC), Montreal, QC H3G 1A4, Canada; 10 Division of Rheumatology, Department of Medicine, Jewish General Hospital, Montreal, QC H3T 1E2, Canada

**Keywords:** vaccines, vaccine hesitancy, COVID-19 infection, immunization, vaccination

## Abstract

We assessed the frequency and correlates of COVID-19 vaccine hesitancy before Canada’s vaccine rollout. A cross-sectional vaccine hesitancy survey was completed by consecutive patients/family members/staff who received the influenza vaccine at McGill University affiliated hospitals. Based on the self-reported likelihood of receiving a future vaccine (scale 0–10), the following three groups were defined: non-hesitant (score 10), mildly hesitant (7.1–9.9), and significantly hesitant (0–7). Factors associated with vaccine hesitancy were assessed with multivariate logistic regression analyses and binomial logistic regression machine learning modelling. The survey was completed by 1793 people. Thirty-seven percent of participants (n = 669) were hesitant (mildly: 315 (17.6%); significantly: 354 (19.7%)). Lower education levels, opposition and uncertainty about vaccines being mandatory, feelings of not receiving enough information about COVID-19 prevention, perceived social pressure to get a future vaccine, vaccine safety concerns, uncertainty regarding the vaccine risk-benefit ratio, and distrust towards pharmaceutical companies were factors associated with vaccine hesitancy. Vaccine safety concerns and opposition to mandatory vaccinations were the strongest correlates of vaccine hesitancy in both the logistic regressions and the machine learning model. In conclusion, in this study, over a third of people immunized for influenza before the COVID-19 vaccine rollout expressed some degree of vaccine hesitancy. Effectively addressing COVID-19 vaccine safety concerns may enhance vaccine uptake.

## 1. Introduction

The coronavirus disease 2019 (COVID-19) pandemic, caused by the severe acute respiratory syndrome coronavirus 2 (SARS-CoV-2), has profoundly impacted our society [1]. Several SARS-CoV-2 vaccines have been demonstrated to be safe and effective at preventing symptomatic infection and COVID-19 related hospitalizations and death [2,3,4,5]. At the time of writing, six of these vaccines have been approved in Canada, including Pfizer-BioNTech, Moderna Spikevax, AstraZeneca Vaxzevria, Janssen (Johnson & Johnson), Medicago Covifenz, and Novavax Nuvaxovid [6]. Despite vaccine availability, vaccine hesitancy is a major threat to vaccine acceptance and uptake.

Vaccine hesitancy is defined as refusal or a delay in acceptance of vaccination, despite the availability of immunization services [7]. This complex and multifactorial worldwide phenomenon is one of the major obstacles to enhancing global health [8]. Vaccine hesitancy has context-specific implications and varies across time, place, and product [7]. In the UK (prior to COVID-19 vaccine rollout), higher levels of hesitancy were present in groups traditionally linked with lower general vaccination rates (e.g., black race/ethnicity, South Asians of Pakistani and Bangladeshi heritage) and varied with education level [9]. The stated reasons for vaccine hesitancy were similar across ethnic groups [9]. However, most of the pre-vaccine rollout studies described expected uptake rates with limited exploration of factors that could potentially influence the acceptance of COVID-19 vaccination. In the current study (i.e., cross-sectional survey), we evaluated COVID-19 vaccine hesitancy among a convenience sample of people receiving the seasonal inactivated influenza vaccine in Nov/Dec 2020. Informed by the framework of the World Health Organization (WHO) Strategic Advisory Group of Experts on Immunization (SAGE) working group [10], we characterized the key factors related to vaccine hesitancy and explored the associations between risk-taking attitudes and COVID-19 vaccine hesitancy. Finally, we built models to identify the factors associated with vaccine hesitancy.

## 2. Materials and Methods

### 2.1. Local Context during the Global Pandemic

Since October 2020, a sharp increase in positive tests for COVID-19 occurred in Quebec and on 12 November 2020, the province announced social gathering restrictions as part of a public health emergency plan (e.g., distancing, masking, etc.). The first COVID-19 vaccines to receive authorization for use in Canada were Pfizer/BioNTech on 9 December and Moderna on 23 December 2020.

### 2.2. Participants

Individuals (n = 2491) who voluntarily agreed, between 2 November and 11 December 2020 (pre-vaccine rollout), to receive the seasonal influenza vaccine to one of the two major adult hospitals of the McGill University Health Centre (MUHC), Montreal General Hospital or Glen Site, were approached to complete the survey. The vaccine clinic was open to all members of the community. Patients were informed about the study by the vaccinating nurse at the time they received the influenza vaccine. No remuneration was offered for completing the survey.

### 2.3. Data Collection Procedure

Adults who consented to this study completed a paper survey in their language of preference (English or French) during the 15 min post-vaccination observation period. The survey was anonymous.

### 2.4. Measures

We assessed the willingness to receive a future COVID-19 vaccine, as well as factors potentially associated with COVID-19 vaccine hesitancy. Those factors were selected from the framework proposed by the World Health Organization (WHO) Strategic Advisory Group of Experts on Immunization (SAGE) working group [10]. In addition, we explored the associations between risk-taking attitudes and COVID-19 vaccine hesitancy. All questions were multiple choice, except for age, which was a free-text field, and willingness to receive a future COVID-19 vaccine, which was measured on a 10-point scale (0 = not likely, 5 = intermediate, and 10 = highly likely; tick-marks every 2.5). The questionnaire was developed in English and then underwent a systematic question-by-question translation to French, with adaptation notes that were reviewed, and then back-translated to ensure congruency and validity. This survey included the following five sections:

- Demographics, including age, sex, race/ethnic minorities associated with increased COVID-19 infection/mortality, role (patient, health care provider, family member, other), employment status, household members, smoking status, and risk factors for severe illness from COVID-19 infection (e.g., medical co-morbidities, treatments).
- Previous seasonal influenza vaccination.
- Previous COVID-19 infection and perception of amount of COVID-19 preventative information received.
- Likelihood of receiving a future vaccine and causes of vaccine hesitancy, using questions proposed by the WHO-SAGE working group adapted to the COVID-19 vaccine [10].
- Likelihood of engaging in risk-associated behaviors based on the adult Domain-Specific Risk-Taking (DOSPERT) health and safety subscale, and medical risk domain add-on [11,12]. A risk-taking score was calculated based on the sum of scores in each question; the sum was transformed to a 0–100 scale (%) and converted to a three-level categorical variable (low, moderate, high risk-taking) by tertiles.

### 2.5. Data Analysis Procedure

We summarized the population characteristics and distributions of responses using frequency distributions for categorical variables and means and standard deviations for continuous variables. Multiple imputation was used for the variables with missing data. The following three groups were defined based upon the frequency distribution of self-reported likelihood of getting a future COVID-19 vaccine: (a) significantly hesitant (values 0–7), (b) mildly hesitant (values 7.1–9.5; there were no participants with hesitancy scores between 9.5 and 9.9), and (c) non-hesitant (value 10). Two multivariate multinomial logistic regression models were built to identify factors associated with significant or mild hesitancy, using non-hesitancy as the reference group. One model included all covariates (saturated model), and another one (parsimonious/partial) featured stepwise backward elimination of the non-significant variables.

A multinomial logistic regression machine learning model was initially built to evaluate how well the survey responses predicted the outcome of significant and mild hesitancy. As a result, binary logistic regression models were built to predict any hesitancy (values 0–9.5). The models were trained using 200 replications of bootstrap sampling with replacement, and performance of each model was evaluated on the unused (out-of-sample or out-of-bag) data and corrected for optimism/overfitting [13,14]. Lasso was also used for shrinkage and selection. Categorical covariates were converted to a numerical format, using one-hot encoding [15]. Each covariate was standardized via z-scores. The discriminative performance of the combined set of non-sample (out-of-sample/out-of-bag) test data was measured using the area under the receiver operating characteristic curve, and the calibration performance was reported using the mean calibration and the calibration slope, with 95% confidence intervals.

All analyses were conducted using SPSS version 24.0 (IBM Corp., Armonk, NY, USA) and Matlab 2019b (The Mathworks, Natick, MA, USA).

## 3. Results

### 3.1. Study Population

A total of 1793 out of 2491 eligible individuals (72%) agreed to complete the survey. The median age of the study participants was 52.2 years (range 18–97 years) (Table 1/Supplementary Table S1: imputed/non-imputed datasets). Two-thirds completed the survey in English (n = 1185; 66.1%). Over half of the participants were females, most were non-smokers, over half reported university education, and almost two-thirds were employed. Almost half of the participants were receiving regular care at the MUHC, and about a third were self-defined health care professionals. About one-fourth reported race/ethnicity associated with increased COVID-19 infection/mortality (i.e., indigenous peoples, black, or Latin American). Twenty percent reported living with a household member at high risk for influenza-related complications (i.e., an adult aged 65 and older, an individual with a chronic illness, or an infant < 6 months old). About 20% of participants were immunosuppressed and 20% had hypertension.

The vast majority had previously received the seasonal influenza vaccine, had never rejected it, and felt they were receiving enough information about COVID-19 prevention. Only 3% had a previous COVID-19 diagnosis (Table 2).

Concerning a future COVID-19 vaccine, over a third of respondents (669/1793) were hesitant, about half (354/669) of whom were significantly hesitant. Most participants trusted the government to make decisions in society’s best interest, while just over half considered that COVID-19 vaccines should be mandatory and had confidence that pharmaceutical companies would provide safe and effective COVID-19 vaccines. Almost a third of participants believed that barriers related to waiting time (at the vaccine provider) and travel (distance, time and effort of traveling, associated costs) might limit their access to a COVID-19 vaccine. Although most participants indicated they were ‘a little’ concerned about the safety of COVID-19 vaccines, over three-quarters considered that in general, vaccine benefits outweigh risks. Only 20% of the individuals anticipated any social pressure to receive a future COVID-19 vaccine. The proportion of participants with high risk-taking behaviors was similar in all hesitancy groups and did not differ between patients and health care professionals.

### 3.2. Factors Associated with COVID-19 Vaccine Hesitancy

The results of the full (Table 3) and parsimonious (Supplementary Table S2) multivariate logistic regression models found positive associations with specific viewpoints, including feelings of not receiving enough information about COVID-19 prevention, opposition/uncertainty about vaccines being mandatory, perceived social pressure, uncertainty regarding the vaccine risk-benefit ratio and vaccine safety concerns. In addition, significantly hesitant participants were more likely to have a lower level of education, to be employed, to have low risk-taking behavior scores and to distrust or be uncertain about trusting pharmaceutical companies to provide safe/effective COVID-19 vaccines.

### 3.3. Prediction of COVID-19 Vaccine Hesitancy

**Figure 1:**
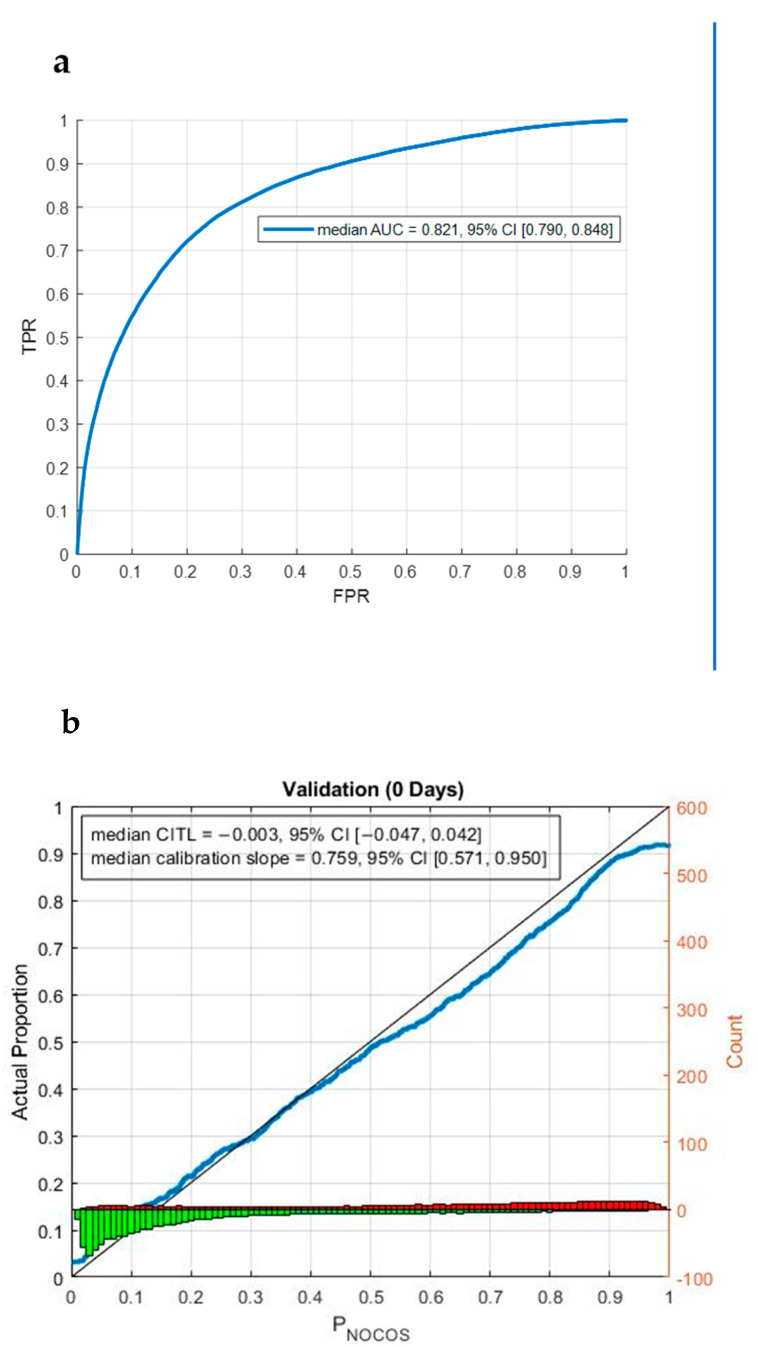
Machine learning model. (**a**) ROC curve discriminative performance of the logistic regression machine learning model comparing no hesitancy with combined mild hesitancy and significant hesitancy. The median AUC and 95% CI are reported; (**b**) calibration performance of the machine learning model. The blue trace is the smoothed probabilities, the black line indicates the ideal calibration, the red and green histograms are the counts of the binned probabilities for vaccine hesitant and non-hesitant patients, respectively (TPR = true positive rate, FPR = false positive rate, AUC = area under the receiver operating characteristic curve, CITL = calibration-in-the-large).

**Figure 2:**
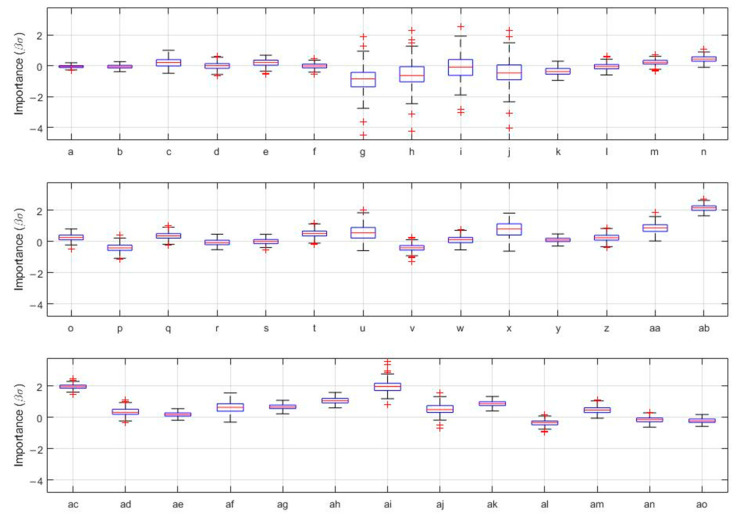
Machine learning models of vaccine hesitancy; boxplots of out-of-sample importance of factors from a single imputation and 200 repetitions of bootstrap resampling for logistic regression models, with the outcome of significant or some hesitancy vs. no hesitancy. ^a^ Age; ^b^ sex (male); ^c^ role (family member); ^d^ role (health care professional), ^e^ role (patient); ^f^ part of a vulnerable population (yes); ^g^ highest level of education (bachelor’s degree or above); ^h^ highest level of education (high school graduate); ^i^ highest level of education (some high school); ^j^ highest level of education (technical/vocational training); ^k^ employment (retired), ^l^ employment (yes); ^m^ living with (healthy adults); ^n^ living with (high risk people); ^o^ living with (susceptible people); ^p^ immunosuppression (yes—other); ^q^ immunosuppression (yes—rheumatic disease); ^r^ cancer (yes); ^s^ hypertension (yes); ^t^ diabetes mellitus (yes); ^u^ kidney disease (yes); ^v^ smoke (yes); ^w^ previous influenza vaccine (yes); ^x^ previous COVID-19 infection (yes); ^y^ language (English); ^z^ previously rejected influenza vaccine (yes); ^aa^ perception of receiving enough information about COVID-19 prevention (no); ^ab^ vaccine compulsory (no); ^ac^ vaccine compulsory (unsure), ^ad^ trust that the government is making decisions in citizens’ best interest regarding COVID-19 vaccines (no); ^ae^ access barrier to vaccination would prevent participants from receiving a COVID-19 vaccine (yes); ^af^ trust pharmaceutical companies to provide safe and effective COVID-19 vaccines (no); ^ag^ trust pharmaceutical companies to provide safe and effective COVID-19 vaccines (unsure); ^ah^ concerns that a future COVID-19 vaccine might not be safe (a little concerned); ^ai^ concerns that a future COVID-19 vaccine might not be safe (very concerned); ^aj^ vaccine benefits outweigh its risks (no); ^ak^ vaccine benefits outweigh its risks (unsure); ^al^ social pressure to receive a COVID-19 vaccine in the future (no); ^am^ social pressure to receive a COVID-19 vaccine in the future (unsure); ^an^ risk-taking behavior (moderate); ^ao^ risk-taking behavior (high).

The full binomial logistic regression machine learning model, including all available variables, showed excellent discriminative performance in terms of being associated with overall hesitancy (area under the curve, AUC 0.82, 95% CI 0.79–0.85) (Figure 1a), and the model was also well calibrated (calibration-in-the-large 0.00, 95% CI −0.05–0.04; calibration slope = 0.76, 95% CI 0.57–0.95) (Figure 1b). This suggests the utility and validity of the model. In the machine learning model, perceptions/uncertainty about COVID-19 vaccines being mandatory and concerns about vaccine safety were the strongest correlates of COVID-19 vaccine hesitancy (Figure 2). An additional model, including only ‘objective’ demographic factors (i.e., excluding patient perceptions), is presented in Supplementary Figures S1 and S2. The performance of this model was inferior to the one that included all variables.

## 4. Discussion

This cross-sectional survey conducted prior to the COVID-19 vaccine rollout in Canada revealed that 20% of people from a convenience sample who voluntarily agreed to influenza vaccination (i.e., likely to represent those who are generally accepting of vaccines) had significant vaccine hesitancy. The strongest factors associated with significant hesitancy included opposition/uncertainty regarding mandatory vaccination, concerns about vaccine safety, uncertainty about the vaccine risk: benefit ratio, lack of trust in pharmaceutical companies, low level of education, and low risk-taking behavior.

The proportion of participants ‘not likely at all’ (score of 0) to receive a COVID-19 vaccine in our study was 2%, while those who were highly likely to accept it (score of 10) represented 63%. In a previous survey that assessed the likelihood of vaccine acceptance in 19 countries, including Canada, 8% of the participants reported they would not accept a future COVID-19 vaccine [16], while 69% of Canadians indicated that they would accept a COVID-19 vaccine if proven safe and effective. Although the anchor question differed, the overall frequency of vaccine acceptance seems to be similar in both studies (63–69%). However, the rate of ‘likely refusal’ was lower in our study, which almost certainly reflects differences in the subjects surveyed (i.e., people who consistently accepted the influenza vaccine before versus the general population). Overall, the rate of hesitancy we found is consistent with evidence from larger international studies (~30% of COVID-19 vaccine hesitancy in the general population) [17].

Over half of the participants in this survey had a university education and were employed. The association between level of education or employment and COVID-19 vaccine hesitancy was inconsistent in previous work. While some studies suggested that employed individuals are more likely to accept a COVID-19 vaccine, others suggest the opposite may be true [18,19,20,21,22]. The fact that some degree of hesitancy was expressed by a third of a socially advantaged population in our study is of concern [9,23].

In the current study, individuals accepting vaccines were more likely to agree with the idea that vaccines should be mandatory, trusted the government to make decisions in society’s best interest, and were confident that pharmaceutical companies would provide safe and effective COVID-19 vaccines. The concept of trust or confidence in the need for/value of vaccines has the strongest association with vaccine uptake [24]. Similar to the hesitancy reported for other vaccines [25,26,27,28], mistrust of either healthcare providers, government, or pharmaceutical companies was associated with less favorable attitudes in our survey. Understanding how trusted sources can effectively harness the tools of social and traditional media to increase knowledge and awareness may help improve COVID-19 vaccine uptake [29].

In this study, vaccine hesitancy was associated with the participant’s impression of not receiving enough information about COVID-19 prevention, disagreement/uncertainty regarding vaccine benefits and risks, and safety related-concerns. This highlights the relevance of developing a multifaceted program of intervention with the sharing of information by trusted sources at its core. Vaccine development and use depend on data-driven assessments of benefits and risks, first by regulatory bodies, and then by individual physicians and patients. Recognizing individual concerns and tailoring the message to specific groups is crucial to positively influence vaccine acceptance [24,30]. We and others have shown that health care professionals are the most trusted source of information and can significantly impact vaccine acceptance [30,31,32,33,34]. In addition, public health bodies are also trusted advisors on COVID-19 vaccines [30]. Disagreement/uncertainty regarding whether COVID-19 vaccination should be compulsory was also associated with COVID-19 vaccine hesitancy. While mandatory vaccination can be ethically justified in the setting of a grave threat to public health, the vaccine(s) offered must be safe and effective, the expected benefit of mandatory vaccination must be greater than other alternatives, and that the level of coercion for non-compliance must be proportionate. In almost all circumstances, encouraging voluntary vaccination is preferred to mandating vaccination [35,36], which may be perceived as a threat to freedom and could undermine public confidence and trust [37,38]. Moreover, for individuals with low a priori vaccination intentions, mandating vaccination could lower uptake of other, still voluntary vaccines and reduce the acceptance of other protective measures targeting the spread of SARS-CoV-2 [39]. Policies aimed at strengthening trust in public health organizations and healthcare systems by addressing specific concerns of unique community subgroups and facilitating access to vaccines should be exhausted before considering a COVID-19 vaccine mandate.

Significant COVID-19 vaccine hesitancy was associated with low risk-taking behaviors. “Risk taking” involves perceptions of expected benefits or harm, with respect to the risk behavior. One’s preference for risky options is assumed to reflect a trade-off between expected benefit or value, and its riskiness (potential for undue cost or harm). To the best of our knowledge, this is the first study to report this finding.

This study has several limitations, which include the following: (1) selection bias, as we only surveyed people who were generally unopposed to vaccines (i.e., they voluntarily visited a seasonal influenza vaccine clinic); (2) the fact that it was limited to two large hospitals from a single university center in an urban area; (3) the lack of validation of the intention to accept or refuse the COVID-19 vaccine with the actual vaccination status; and, (4) the risk of reporting bias, given that the data were self-reported. Despite the study’s limitations, the fact that these data were collected in a ‘vaccine-favorable environment’ not only highlights the magnitude of the problem but, by identifying the strongest correlates of vaccine hesitancy, the findings provide potentially actionable targets for interventions to enhance vaccine uptake. Our findings are also interesting, as they included both healthcare workers and patients.

## 5. Conclusions

We found significant COVID-19 vaccine hesitancy to be present even among a group of people who sought out the influenza vaccine. Multiple factors were associated with this phenomenon, but those most strongly associated were disagreement/uncertainty that COVID-19 vaccines should be mandatory, safety concerns, doubts about benefits versus risks, and not trusting pharmaceutical companies. Carefully targeted educational interventions, focusing on the advantages and safety of COVID-19 vaccines, delivered by individuals and organizations most trusted by people (e.g., healthcare providers, leaders in healthcare and in the workforce, and government public health representatives) may promote vaccine uptake and reduce COVID-19 vaccine hesitancy.

## Figures and Tables

**Table 1:** Participants’ characteristics according to their likelihood to receive a COVID-19 vaccine.

Variables	No Hesitancy (10) (n = 1124)	Mild Hesitancy (7.1–9.5 *) (n = 315)	Significant Hesitancy (0–7) (n = 354)	Total (n = 1793)
	n (%) or mean ± SD	n (%) or mean ± SD	n (%) or mean ± SD	n (%) or mean ± SD
**Demographics**				
**Age**	53.2 ± 17.0	49.9 ± 16.9	50.8 ± 16.2	52.2 ± 16.9
**Sex**	‘**Are you a:**’ options: female, male			
Female	628 (55.9)	186 (59.0)	237 (66.9)	1051 (58.6)
Male	496 (44.1)	129 (41.0)	117 (33.1)	742 (41.4)
**Role**	‘**Are you a:**’ options provided as listed below.			
Patient	512 (45.6)	133 (42.2)	175 (49.4)	820 (45.7)
Healthcare professional	337 (30.0)	108 (34.3)	97 (27.4)	542 (30.2)
Family member	137 (12.2)	42 (13.3)	49 (13.8)	228 (12.7)
Other ^a^	138 (12.3)	32 (10.2)	33 (9.3)	203 (11.3)
**Vulnerable population ^b^**	‘**Are you:**’ options: indigenous people, Latin, black			
Yes	228 (20.3)	75 (23.8)	113 (31.9)	416 (23.2)
No	896 (79.7)	240 (76.2)	241 (68.1)	1377 (76.8)
**Education**	‘**What is the highest degree or level of school you have completed?**’			
Some high school education or less	58 (5.2)	19 (6.0)	29 (8.2)	106 (5.9)
High school graduate	154 (13.7)	41 (13.0)	65 (18.4)	260 (14.5)
Technical/vocational training	239 (21.3)	87 (27.6)	93 (26.3)	419 (23.4)
Bachelor’s degree or above	673 (59.9)	168 (53.3)	167 (47.2)	1008 (56.2)
**Employment**	‘**Are you currently employed?**’			
No	166 (14.8)	52 (16.5)	69 (19.5)	287 (16.0)
Yes	682 (60.7)	206 (65.4)	228 (64.4)	1116 (62.2)
Retired	276 (24.6)	57 (18.1)	57 (16.1)	390 (21.8)
**Living…**	‘**With whom do you live? Please indicate all options that correspond**’			
With high-risk people ^c^	237 (21.1)	78 (24.8)	109 (30.8)	424 (23.6)
With susceptible people ^d^	231 (20.6)	55 (17.5)	67 (18.9)	353 (19.7)
With healthy adults	382 (34.0)	119 (37.8)	110 (31.1)	611 (34.1)
Alone	274 (24.4)	63 (20.0)	68 (19.2)	405 (22.6)
**Immunosuppressed**	‘**Please indicate if you are diagnosed with any of the following conditions/currently taking any of these medications (options)**’			
Yes (other ^e^)	117 (10.4)	24 (7.6)	28 (7.9)	169 (9.4)
Yes (rheumatic disease)	106 (9.4)	34 (10.8)	51 (14.4)	191 (10.7)
No	901 (80.2)	257 (81.6)	275 (77.7)	1433 (79.9)
**Cancer**				
Yes	176 (15.7)	45 (14.3)	49 (13.8)	270 (15.1)
No	948 (84.3)	270 (85.7)	305 (86.2)	1523 (84.9)
**Hypertension**				
Yes	231 (20.6)	65 (20.6)	66 (18.6)	362 (20.2)
No	893 (79.4)	250 (79.4)	288 (81.4)	1431 (79.8)
**Diabetes mellitus**				
Yes	114 (10.1)	36 (11.4)	43 (12.1)	193 (10.8)
No	1010 (89.9)	279 (88.6)	311 (87.9)	1600 (89.2)
**Kidney disease**				
Yes	16 (1.4)	8 (2.5)	5 (1.4)	29 (1.6)
No	1108 (98.6)	307 (97.5)	349 (98.6)	1764 (98.4)
**Smoking**				
Yes	94 (8.4)	26 (8.3)	32 (9.0)	152 (8.5)
No	1030 (91.6)	289 (91.7)	322 (91.0)	1641 (91.5)
**Language ^f^**				
English	723 (64.3)	204 (64.8)	258 (72.9)	1185 (66.1)
French	401 (35.7)	111 (35.2)	96 (27.1)	608 (33.9)

* There were no participants with a hesitancy score between 9.5 and 9.9; ^a^ other non-professional health care workers, researchers, students, and volunteers; ^b^ indigenous people, black (African American, Caribbean), Latin; ^c^ child who goes to elementary school or young adult; ^d^ adult older than 65 years, individual with chronic disease, or infant <6 months old; ^e^ HIV, inflammatory bowel disease, cancer, and transplant; ^f^ of the survey—participants had two options, English or French.

**Table 2:** Vaccination-related factors according to participants’ likelihood to receive a COVID-19 vaccine.

Variables	No Hesitancy (10) (n = 1124)	Mild Hesitancy (7.1–9.5 *) (n = 315)	Significant Hesitancy (0–7) (n = 354)	Total (n = 1793)
	n (%)	n (%)	n (%)	n (%)
**Influenza vaccine status**				
**Previous influenza vaccine**	‘Have you **ever received a FLU vaccine before**?’			
Yes	1017 (90.5)	280 (88.9)	299 (84.5)	1596 (89.0)
No	107 (9.5)	35 (11.1)	55 (15.5)	197 (11.0)
**Previously rejected influenza vaccine**	‘Have you **ever rejected the FLU vaccine**?’			
Yes	57 (5.1)	21 (6.7)	35 (9.9)	113 (6.3)
No	1067 (94.9)	294 (93.3)	319 (90.1)	1680 (93.7)
**COVID-19 disease**				
**Previous COVID-19 diagnosis**	‘Were you **diagnosed with COVID-19** in 2020?’			
Yes	23 (2.0)	11 (3.5)	10 (2.8)	44 (2.5)
No	1101 (98.0)	304 (96.5)	344 (97.2)	1749 (97.5)
**Perception that they receive enough information about COVID-19 prevention**	‘**Do you feel you get enough information about COVID-19 prevention?**’			
No	36 (3.2)	28 (8.9)	31 (8.8)	95 (5.3)
Yes	1088 (96.8)	287 (91.1)	323 (91.2)	1698 (94.7)
**COVID-19 vaccine**				
**COVID-19 vaccine compulsory**	‘**Do you think the COVID-19 vaccine should be compulsory?**’			
No	78 (6.9)	40 (12.7)	99 (28.0)	217 (12.1)
Unsure	202 (18.0)	163 (51.7)	200 (56.5)	565 (31.5)
Yes	844 (75.1)	112 (35.6)	55 (15.5)	1011 (56.4)
**Trust that the government is making decisions in citizens’ best interest regarding COVID-19 vaccines**	‘**Do you trust that our government is making decisions in our best interest with respect to the COVID-19 vaccine?**’			
No	68 (6.0)	22 (7.0)	75 (21.2)	165 (9.2)
Yes	1056 (94.0)	293 (93.0)	279 (78.8)	1628 (90.8)
**Access barrier to vaccination would prevent participants from receiving a COVID-19 vaccine ^a^**	‘**Would any of the following factors prevent you from getting the COVID-19 vaccine? (*please indicate all that correspond*)**’ **^a^**			
Yes	277 (24.6)	106 (33.7)	130 (36.7)	513 (28.6)
No	847 (75.4)	209 (66.3)	224 (63.3)	1280 (71.4)
**Trust pharmaceutical companies in providing safe and effective COVID-19 vaccines**	‘**Do you trust pharmaceutical companies to provide safe and effective COVID-19 vaccines?**’			
No	25 (2.2)	17 (5.4)	47 (13.3)	89 (5.0)
Unsure	339 (30.2)	161 (51.1)	240 (67.8)	740 (41.3)
Yes	760 (67.6)	137 (43.5)	67 (18.9)	964 (53.8)
**Concerns that a future COVID-19 vaccine might not be safe**	‘**How concerned are you that a future COVID-19 vaccine might not be safe?**’			
Very concerned *(to the point I will refuse to get it)*	27 (2.4)	13 (4.1)	83 (23.4)	123 (6.9)
A little concerned	695 (61.8)	270 (85.7)	241 (68.1)	1206 (67.3)
Not concerned at all	402 (35.8)	32 (10.2)	30 (8.5)	464 (25.9)
**Vaccine benefits overweigh its risks**	‘**Do you think that vaccine benefits, in general, are larger than their risks?**’			
No	33 (2.9)	2 (0.6)	19 (5.4)	54 (3.0)
Unsure	103 (9.2)	76 (24.1)	174 (49.2)	353 (19.7)
Yes	988 (87.9)	237 (75.2)	161 (45.5)	1386 (77.3)
**Social pressure to receive a COVID-19 vaccine in the future**	‘**Do you feel social pressure to get a COVID-19 vaccine in the future?**’			
Yes	205 (18.2)	86 (27.3)	75 (21.2)	366 (20.4)
Unsure	103 (9.2)	61 (19.4)	89 (25.1)	253 (14.1)
No	816 (72.6)	168 (53.3)	190 (53.7)	1174 (65.5)
**Risk-taking behaviour ^**				
Low (≤26.67)	378 (33.6)	105 (33.3)	151 (42.7)	634 (35.4)
Moderate (26.68–38.33)	317 (28.2)	91 (28.9)	95 (26.8)	503 (28.1)
High (≥38.34)	429 (38.2)	119 (37.8)	108 (30.5)	656 (36.6)

* There were no participants with a hesitancy score between 9.5 and 9.9; ^a^ distance to the vaccine provider, time needed to get to the vaccine provider, waiting time at the vaccine provider, cost/parking getting to the vaccine provider, and effort of traveling to the vaccine provider; ^ statements on risk-taking are in Supplementary Information (page 3–4 of survey).

**Table 3:** Multivariate analysis of factors associated with vaccine hesitancy (non-hesitancy as reference group).

Level of Hesitancy to Receive a COVID-19 Vaccine	Significant Hesitancy (Score 0–7)	Mild Hesitancy (Scores 7.1–9.5)
	aOR * (95% CI) (df, Wald, *p*-Value)	
**Age**	1.00 (0.99–1.02) (1, 0.14, 0.712)	1.00 (0.98–1.01) (1, 0.75, 0.387)
**Employment**		
No	1.77 (0.96–3.25) (1, 3.39, 0.066)	1.30 (0.76–2.21) (1, 0.92, 0.338)
Yes	**1.72 (1.00–2.97**) (1, 3.86, **0.049**)	1.17 (0.73–1.86) (1, 0.43, 0.512)
Retired	Ref	Ref
**Sex**		
Female	1.29 (0.91–1.83) (1, 1.99, 0.158)	0.95 (0.70–1.28) (1, 0.13, 0.723)
Male	Ref	Ref
**Role**		
Patient	1.21 (0.68–2.17) (1, 0.43, 0.514)	1.13 (0.68–1.88) (1, 0.22, 0.640)
Healthcare professional	0.75 (0.41–1.36) (1, 0.91, 0.342)	1.15 (0.70–1.91) (1, 0.31, 0.578)
Family member	1.02 (0.51–2.02) (1, 0.002, 0.966)	1.17 (0.64–2.12) (1, 0.25, 0.614)
Other	Ref	Ref
**Vulnerable Population ^a^**		
Yes	1.32 (0.91–1.92) (1, 2.07, 0.151)	1.02 (0.72–1.44) (1, 0.01, 0.919)
No	Ref	Ref
**Education**		
Some high-school education or less	**3.57 (1.81–7.07)** (1, 13.41, **<0.001**)	1.85 (0.97–3.53) (1, 3.53, 0.060)
High-school graduate	**1.70 (1.04–2.78)** (1, 4.48, **0.034**)	1.22 (0.78–1.91) (1, 0.76, 0.384)
Technical/vocational training	**1.54 (1.03–2.30)** (1, 4.44, **0.035**)	**1.50 (1.07–2.13)** (1, 5.37, **0.020**)
Bachelor’s degree or above	Ref	Ref
**Living…**		
With high-risk people ^b^	1.52 (0.93–2.48) (1, 2.75, 0.097)	1.40 (0.91–2.16) (1, 2.31, 0.128)
With susceptible people ^c^	1.40 (0.83–2.35) (1, 1.58, 0.209)	1.20 (0.75–1.90) (1, 0.58, 0.448)
With healthy adults	0.99 (0.63–1.58) (1, 0.001, 0.981)	1.36 (0.92–2.02) (1, 2.37, 0.124)
Alone	Ref	Ref
**Immunosuppressed**		
Yes—other ^d^	0.64 (0.33–1.24) (1, 1.77, 0.184)	0.67 (0.38–1.19) (1, 1.89, 0.170)
Yes—rheumatic disease	1.42 (0.83–2.41) (1, 1.66, 0.197)	1.22 (0.75–1.99) (1, 0.63, 0.429)
No	Ref	Ref
**Cancer**		
Yes	0.81 (0.48–1.36) (1, 0.63, 0.426)	0.97 (0.62–1.54) (1, 0.01, 0.907)
No	Ref	Ref
**Hypertension**		
Yes	0.77 (0.49–1.21) (1, 1.26, 0.261)	1.12 (0.76–1.64) (1, 0.32, 0.571)
No	Ref	Ref
**Diabetes mellitus**		
Yes	1.60 (0.90–2.82) (1, 2.57, 0.109)	1.57 (0.97–2.54) (1, 3.40, 0.065)
No	Ref	Ref
**Kidney disease**		
Yes	1.08 (0.31–3.71) (1, 0.01, 0.908)	2.33 (0.87–6.22) (1, 2.82, 0.093)
No	Ref	Ref
**Smoking**		
Yes	0.66 (0.37–1.18) (1, 1.96, 0.162)	0.81 (0.49–1.36) (1, 0.62, 0.430)
No	Ref	Ref
**Previous influenza vaccine**		
Yes	0.99 (0.61–1.63) (1, 0.001, 0.982)	1.04 (0.66–1.66) (1, 0.03, 0.857)
No	Ref	Ref
**Previously rejected influenza vaccine**		
Yes	1.33 (0.70–2.50) (1, 0.76, 0.383)	1.14 (0.64–2.04) (1, 0.20, 0.659)
No	Ref	Ref
**Previous COVID-19 diagnosis**		
Yes	2.04 (0.76–5.50) (1, 2.01, 0.157)	1.91 (0.82–4.44) (1, 2.26, 0.133)
No	Ref	Ref
**Perception of receiving enough information about COVID-19 prevention**		
No	**2.20 (1.10–4.40)** (1, 4.91, **0.027**)	**2.82 (1.55–5.11)** (1, 11.65, **0.001**)
Yes	Ref	Ref
**COVID-19 vaccine compulsory**		
No	**19.76 (11.82–33.06)** (1, 129.23, **<0.001**)	**3.98 (2.50–6.34)** (1, 33.69, **<0.001**)
Unsure	**12.14 (8.03–18.35)** (1, 140.28, **<0.001**)	**4.91 (3.57–6.74)** (1, 95.60, **<0.001**)
Yes	Ref	Ref
**Trust that the government is making decisions in citizens’ best interest regarding COVID-19 vaccines**		
No	1.67 (0.98–2.82) (1, 3.59, 0.058)	0.82 (0.47–1.44) (1, 0.48, 0.487)
Yes	Ref	Ref
**Access barrier to vaccination would prevent participants from receiving a COVID-19 vaccine ^e^**		
Yes	1.30 (0.92–1.84) (1, 2.14, 0.144)	1.15 (0.84–1.57) (1, 0.75, 0.387)
No	Ref	Ref
**Trust pharmaceutical companies to provide safe and effective COVID-19 vaccines**		
No	**3.75 (1.82–7.73)** (1, 12.78, **<0.001**)	1.86 (0.91–3.81) (1, 2.87, 0.090)
Unsure	**3.06 (2.10–4.45)** (1, 34.28, **<0.001**)	1.32 (0.97–1.79) (1, 3.09, 0.079)
Yes	Ref	Ref
**Concerns that a future COVID-19 vaccine might not be safe**		
Very concerned	**7.02 (3.30–14.94)** (1, 25.56, **<0.001**)	**2.59 (1.12–5.97)** (1, 4.95, **0.026**)
A little concerned	**1.93 (1.16–3.22)** (1, 6.39, **0.011**)	**3.15 (2.06–4.81)** (1, 27.91, **<0.001**)
Not concerned at all	Ref	Ref
**Vaccine benefits outweigh its risks**		
No	**2.95 (1.28–6.79)** (1, 6.49, **0.011**)	0.29 (0.66–1.28) (1, 2.66, 0.103)
Unsure	**3.81 (2.55–5.68)** (1, 42.93, **<0.001**)	**1.74 (1.17–2.58)** (1, 7.45, **0.006**)
Yes	Ref	Ref
**Social pressure to receive a COVID-19 vaccine in the future**		
Yes	1.16 (0.76–1.77) (1, 0.46, 0.500)	**1.76 (1.24–2.50)** (1, 9.98, **0.002**)
Unsure	**2.27 (1.45–3.56)** (1, 12.78, **<0.001**)	**2.11 (1.39–3.19)** (1, 12.47, **<0.001**)
No	Ref	Ref
**Language**		
English	1.37 (0.95–1.98) (1, 2.78, 0.096)	1.02 (0.75–1.40) (1, 0.02, 0.879)
French	Ref	Ref
**Risk-taking behavior**		
Low	**1.60 (1.07–2.40)** (1, 5.26, **0.022**)	1.08 (0.76–1.53) (1, 0.16, 0.686)
Moderate	1.16 (0.76–1.77) (1, 0.46, 0.498)	1.03 (0.72–1.46) (1, 0.02, 0.894)
High	Ref	Ref

* aOR: Adjusted odds ratio. Adjustment was carried out for all variables shown in the table. ^a^ Indigenous people, African American and Latin; ^b^ child who goes to elementary school or young adult; ^c^ adult older than 65 years, individual with chronic disease, or infant < 6 months old. ^d^ HIV, inflammatory bowel disease, cancer, and transplant; ^e^ distance to the vaccine provider, time needed to get to the vaccine provider, waiting time at the vaccine provider, cost/parking getting to the vaccine provider, and effort of traveling to the vaccine provider.

## Data Availability

The data presented in this study are available upon request from the corresponding author.

## Supplementary Materials

The following supporting information can be downloaded at: https://www.mdpi.com/article/10.3390/vaccines10091514/s1, (i) Supplementary Table S1: Characteristics of patients by type of dataset (imputed vs. non-Imputed); (ii) Supplementary Table S2: Multivariate analysis of factors associated with vaccine hesitancy (non-hesitancy as reference group)—reduced/parsimonious model (backward elimination); (iii) Supplementary Figure S1: Machine learning model (including only demographic information); (iv) Supplementary Figure S2: Machine learning correlates of vaccine hesitancy (including only demographic information).
