# Supplementary material for: High Frequency of COVID-19 Vaccine Hesitancy among Canadians Immunized for Influenza: A Cross-Sectional Survey
Source: Vaccines (Basel). 2022 Sep 10;10(9):1514. doi: 10.3390/vaccines10091514 (PMC9506006; doi:10.3390/vaccines10091514)
Supplement: Supplementary file 1 [file vaccines-10-01514-s001.zip › vaccines-1885445-supplementary.pdf]

Supplementary Data

Supplementary Table S1. Characteristics of patients by type of dataset (Imputed vs. Non-Imputed)

Variables	Imputed[a] (N=1,793)	Non-Imputed[b] (N=1,793)
	n (%) or mean ± SD	n (%) or mean ± SD
**Age**	52.2 ± 16.9	52.2 ± 16.9
**Sex**		
Female	1051 (58.6)	1024 (58.5)
Male	742 (41.4)	727 (41.5)
**Role**		
Patient	820 (45.7)	795(45.8)
Healthcare professional	542 (30.2)	524 (30.2)
Family Member	228 (12.7)	220 (12.7)
Other	203 (11.3)	195 (11.2)
**Vulnerable population[c]**		
No	1377 (76.8)	1332 (85.1)
Yes	416 (23.2)	234 (14.9)
**Education**		
Some High-School or Less	106 (5.9)	106 (6.0)
High-School Graduate	260 (14.5)	258 (14.5)
Technical/Vocational Training	419 (23.4)	412 (23.2)
Bachelor's Degree or Above	1008 (56.2)	1000 (56.3)
**Employment**		
No	287 (16.0)	286 (16.1)
Yes	1116 (62.2)	1102 (62.0)
Retired	390 (21.8)	389 (21.9)
**Living…**		
Alone	405 (22.6)	370 (21.1)
With healthy adults	611 (34.1)	611 (34.8)
With susceptible people[d]	353 (19.7)	350 (19.9)
With high-risk people[e]	424 (23.6)	424 (24.2)
**Immunosuppressed**		
No	1433 (79.9)	1429 (80.5)
Yes - rheumatic disease	191 (10.7)	183 (10.3)
Yes - other[f]	169 (9.4)	163 (9.2)
**Cancer**		
No	1523 (84.9)	1517 (85.0)

Yes	270 (15.1)	268 (15.0)
**Hypertension**		
No	1431 (79.8)	1420 (80.5)
Yes	362 (20.2)	344 (19.5)
**Diabetes mellitus**		
No	1600 (89.2)	1593 (90.4)
Yes	193 (10.8)	169 (9.6)
**Kidney disease**		
No	1764 (98.4)	1756 (98.4)
Yes	29 (1.6)	29 (1.6)
**Smoking**		
No	1641 (91.5)	1623 (91.5)
Yes	152 (8.5)	150 (8.5)
**Previous influenza vaccine**		
No	197 (11.0)	192 (10.8)
Yes	1596 (89.0)	1588 (89.2)
**Previously rejected influenza vaccine**		
No	1680 (93.7)	1649 (93.9)
Yes	113 (6.3)	108 (6.1)
**Previous COVID-19 diagnosis**		
No	1749 (97.5)	1741 (97.5)
Yes	44 (2.5)	44 (2.5)
**Perception of receiving enough information about COVID-19 prevention**		
No	95 (5.3)	89 (5.0)
Yes	1698 (94.7)	1689 (95.0)
**COVID-19 vaccine compulsory**		
No	217 (12.1)	215 (12.2)
Yes	1011 (56.4)	995 (56.4)
Not Sure	565 (31.5)	555 (31.4)
**Trust that the government is making decisions in citizens' best interest regarding COVID-19 vaccines**		
No	165 (9.2)	146 (8.6)
Yes	1628 (90.8)	1557 (91.4)
**Access barrier to vaccination would prevent participants from receiving a COVID-19 vaccine**[g]		
No	1280 (71.4)	1246 (76.2)
Yes	513 (28.6)	389 (23.8)

**Trust pharmaceutical companies to provide safe and effective COVID-19 vaccines**		
No	89 (5.0)	81 (4.7)
Yes	964 (53.8)	937 (54.0)
Not Sure	740 (41.3)	718 (41.4)
**Concerns that a future COVID-19 vaccine might not be safe**		
Not concerned at all	464 (25.9)	437 (25.2)
A little concerned	1206 (67.3)	1185 (68.4)
Very concerned	123 (6.9)	110 (6.4)
**Vaccine benefits outweigh its risks**		
No	54 (3.0)	47 (2.7)
Yes	1386 (77.3)	1364 (78.6)
Not Sure	353 (19.7)	324 (18.7)
**Social pressure to receive a COVID-19 vaccine in the future**		
No	1174 (65.5)	1153 (66.3)
Yes	366 (20.4)	349 (20.1)
Not Sure	253 (14.1)	238 (13.7)
**Language**		
English	1185 (66.1)	1185 (66.1)
French	608 (33.9)	608 (33.9)
**Risk-taking behaviour**		
Low (≤26.67)	634 (35.4)	580 (33.4)
Moderate (26.68 - 38.33)	503 (28.1)	503 (28.9)
High (≥38.34)	656 (36.6)	655 (37.7)

[a]Missing data were imputed using multiple imputation
[b]Results presented for individuals with available data
[c]Indigenous people, African American and Latin
[d]Adult older than 65 years, individual with chronic disease, or infant <6 months old.
[e]Child who goes to elementary school or young adult
[f]HIV, inflammatory bowel disease, cancer, and transplant
[g]Distance to the vaccine provider, time needed to get to the vaccine provider, waiting time at the vaccine provider, cost/parking in getting to the vaccine provider, and effort of traveling to the vaccine provider

Supplementary Table S2. Multivariate analysis of factors associated with vaccine hesitancy (non-hesitancy as reference group) – Reduced/Parsimonious Model (Backward Elimination)

Level of Hesitancy to Receive COVID-19 Vaccine	aOR* (95%CI)
**Significant hesitant (scores 0-7)**	
**Education**	
Some high school or less	**3.17 (1.66 - 6.07)**
High school graduate	**1.77 (1.11 - 2.83)**
Technical/vocational training	**1.48 (1.00 - 2.18)**
Bachelor's degree or above	Ref
**Living with…**	
Young children	**1.67 (1.05 -2.65)**
Susceptible people[a]	1.38 (0.83 - 2.27)
Healthy adults	1.03 (0.66 - 1.60)
Alone	Ref
**Immunosuppressed**	
Yes – other[b]	0.70 (0.39 - 1.25)
Yes – rheumatic disease	**1.73 (1.06 - 2.81)**
No	.
**Diabetes mellitus**	
Yes	1.52 (0.89 - 2.60)
No	Ref
**Perception of receiving enough information about COVID-19 prevention**	
No	**2.33 (1.18 - 4.61)**
Yes	Ref
**COVID-19 vaccine compulsory**	
No	**18.22 (11.07 - 29.99)**
Unsure	**12.03 (8.06 -17.94)**
Yes	Ref
**Trust that the government is making decisions in citizens' best interest regarding COVID-19 vaccines**	
No	1.59 (0.95 - 2.66)
Yes	Ref
**Trust pharmaceutical companies to provide safe and effective COVID-19 vaccines**	
No	**4.16 (2.03 - 8.53)**
Unsure	**3.13 (2.17 - 4.51)**

Yes	Ref
**Concerns that a future COVID-19 vaccine might not be safe**	
Very concerned	**7.39 (3.51 -15.54)**
A little concerned	**2.01 (1.22 - 3.31)**
Not concerned at all	Ref
**Vaccine benefits outweigh its risks**	
No	**3.31 (1.46 - 7.52)**
Unsure	**4.12 (2.80 - 6.06)**
Yes	Ref
**Social pressure to receive a COVID-19 vaccine in the future**	
Yes	1.18 (0.78 - 1.78)
Unsure	**2.24 (1.45 - 3.48)**
No	Ref
**Mild hesitancy (scores 7.1-9.5)**	
**Education**	
Some high school or less	1.76 (0.95 - 3.26)
High school graduate	1.19 (0.77 - 1.83)
Technical/vocational training	**1.46 (1.04 - 2.05)**
Bachelor's degree or above	Ref
**Living with…**	
Young children	1.48 (0.98 -2.24)
Susceptible people[a]	1.17 (0.75 -1.83)
Healthy adults	1.38 (0.94 - 2.01)
Alone	Ref
**Immunosuppressed**	
Yes – other[b]	0.71 (0.42 -1.18)
Yes – rheumatic disease	1.20 (0.76 -1.90)
No	Ref
**Diabetes mellitus**	
Yes	1.60 (1.01 - 2.52)
No	Ref
**Perception of receiving enough information about COVID-19 prevention**	
No	**2.85 (1.59 - 5.11)**
Yes	Ref
**COVID-19 vaccine compulsory**	
No	**4.12 (2.61 - 6.52)**
Unsure	**4.99 (3.65 - 6.82)**
Yes	Ref

**Trust that the government is making decisions in citizens' best interest regarding COVID-19 vaccines**	
No	0.80 (0.46 - 1.40)
Yes	Ref
**Trust pharmaceutical companies to provide safe and effective COVID-19 vaccines**	
No	1.84 (0.90 -3.74
Unsure	**1.36 (1.00 - 1.84)**
Yes	Ref
**Concerns that a future COVID-19 vaccine might not be safe**	
Very concerned	**2.68 (1.17 - 6.11)**
A little concerned	**3.05 (2.01 - 4.64)**
Not concerned at all	Ref
**Vaccine benefits outweigh its risks**	
No	0.30 (0.07 - 1.31)
Unsure	**1.73 (1.18 - 2.54)**
Yes	Ref
**Social pressure to receive a COVID-19 vaccine in the future**	
Yes	**1.83 (1.31 - 2.57)**
Unsure	**2.14 (1.42 - 3.21)**
No	Ref

*aOR: Adjusted Odds Ratio. Adjustment was done for all variables shown in the table. Variable selection was done using stepwise backward elimination

[a]Adult older than 65 years, individual with chronic disease, or infant <6 months old.

[b]HIV, inflammatory bowel disease, cancer, and transplant

Supplementary Figure S1. Machine learning model (including only demographic information).

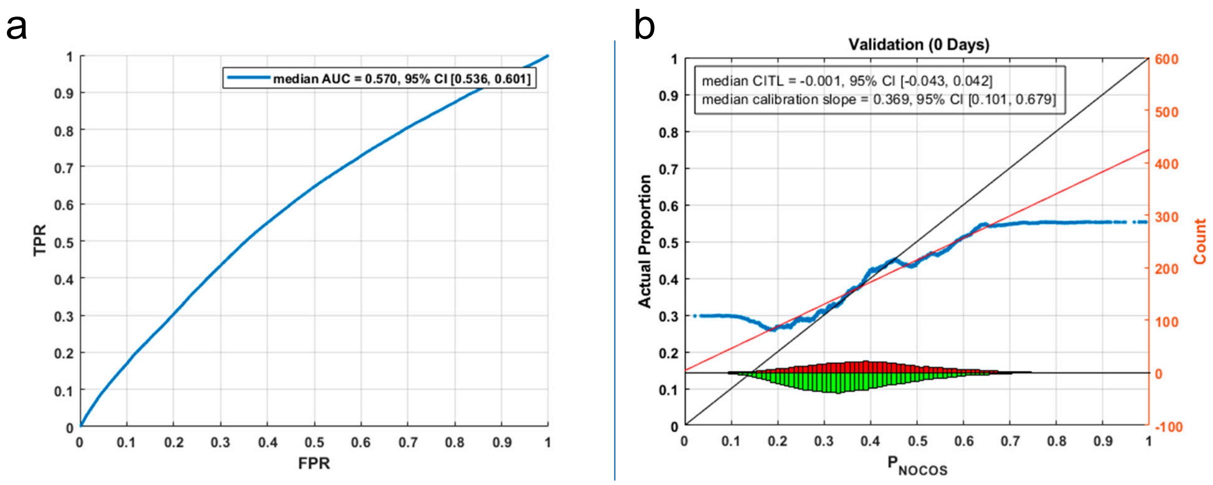

(a) ROC curve discriminative performance of the logistic regression machine learning model comparing no hesitancy with combined mild hesitancy and significant hesitancy. The median AUC and 95% CI is reported; **(b)** Calibration performance of the machine learning model.

Supplementary Figure S2. Machine learning correlates of vaccine hesitancy (including only demographic information).

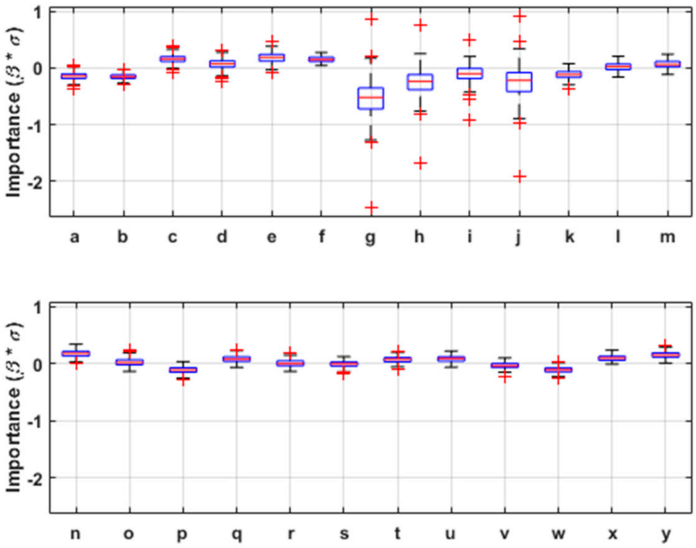

Boxplots of out-of-sample factor importance (adjusted odds ratio * standard deviation) from a single imputation and 200 repetitions of bootstrap resampling for logistic regression models that correlate with significant or some hesitancy vs. no hesitancy.

[a]Age; [b]Sex (male); [c]Role (family member); [d]Role (health care professional), [e]Role (patient); [f]Part of a vulnerable population (yes); [g]Highest level of education (bachelor's degree or above); [h]Highest level of education (high school graduate); [i]Highest level of education (some high school); [j]Highest level of education (technical/vocational training); [k]Employment (retired), [l]Employment (yes); [m]Living with (healthy adults); [n]Living with (high risk people); [o]Living with (susceptible people); [p]Immunosuppression (yes - other); [q]Immunosuppression (yes - rheumatic disease); [r]Cancer (yes); [s]Hypertension (yes); [t]Diabetes mellitus (yes); [u]Kidney disease (yes); [v]Smoke (yes); [w]Previous influenza vaccine (yes); [x]Previous COVID-19 infection (yes); [y]Language (English)
